# Cell-free mitochondrial DNA in progressive multiple sclerosis

**DOI:** 10.1016/j.mito.2018.07.008

**Published:** 2019-05

**Authors:** Hannah Lowes, Angela Pyle, Martin Duddy, Gavin Hudson

**Affiliations:** a Institute of Genetic Medicine, International Centre for Life, Central Parkway, Newcastle upon Tyne NE1 3BZ, UK; b The Wellcome Centre for Mitochondrial Research, Newcastle University, Medical School, Framlington Place, Newcastle upon Tyne NE2 4HH, UK; c Royal Victoria Infirmary, Newcastle-upon-Tyne, UK.

**Keywords:** Cell-free mitochondrial DNA, Progressive Multiple Sclerosis, Next-Generation Sequencing, Neurodegeneration

## Abstract

Recent studies have linked cell-free mitochondrial DNA (ccf-mtDNA) to neurodegeneration in both Alzheimer's and Parkinson's disease, raising the possibility that the same phenomenon could be seen in other diseases which manifest a neurodegenerative component. Here, we assessed the role of circulating cell-free mitochondrial DNA (ccf-mtDNA) in end-stage progressive multiple sclerosis (PMS), where neurodegeneration is evident, contrasting both ventricular cerebral spinal fluid ccf-mtDNA abundance and integrity between PMS cases and controls, and correlating ccf-mtDNA levels to known protein markers of neurodegeneration and PMS. Our data indicate that reduced ccf-mtDNA is a component of PMS, concluding that it may indeed be a hallmark of broader neurodegeneration.

## Highlights

• Cell-free-mtDNA is reduced in the ventricular CSF of progressive multiple sclerosis patients.
• S100β appears elevated in the CSF of progressive multiple sclerosis patients compared to controls.
• Cell-free mtDNA in the ventricular CSF appears largely intact, but does appear to harbour an increase in non-synonymous variation.

## 1 Introduction

Progressive multiple sclerosis (PMS) is a chronic, inflammatory, demyelinating disorder with progressive neurodegeneration (Stadelmann, 2011). Inflammation is an inducer of neuronal damage which, with chronic demyelination, leads to loss of central nervous system (CNS) tissue. White matter demyelination is a cardinal hallmark of PMS, although neuropathological studies also indicate extensive axonal loss in the spinal cord tracts and synapses of the gray matter (Bjartmar et al., 2003; Jurgens et al., 2016).

MS has an established link to mitochondrial dysfunction (Witte et al., 2014). At the cellular level, mitochondrial dysfunction plays a direct role in demyelination, with mitochondrial structural changes, altered gene expression and mitochondrial enzyme activities observed in MS patients (Mao and Reddy, 2010a). At the genetic level, inherited mitochondrial DNA (mtDNA) variation modulates MS risk (Andalib et al., 2013), with mtDNA haplogroups (distinct monophyletic clades of mtDNA variants) both increasing (haplogroup J) (Tranah et al., 2015) and decreasing (haplogroup K) (Hudson et al., 2014) the risk of developing MS. Multiple deletions of mtDNA, typically indicative of primary mitochondrial disease, can be detected in the gray matter of MS cases (Campbell et al., 2011).

In mammals, mtDNA appears more resistant to degradation by nucleases than nDNA (Manuelidis, 2011) and appears to persist in extracellular fluids as circulating, cell-free, mtDNA (ccf-mtDNA). ccf-mtDNA appears associated with neurodegeneration, with significant decreases in ccf-mtDNA observed in the cerebrospinal fluid (CSF) of patients with Parkinson's disease (PD) (Pyle et al., 2015a) and Alzheimer's disease (AD) (Podlesniy et al., 2013). Conversely, elevated CSF ccf-mtDNA appears associated with traumatic brain injury in children (Podlesniy et al., 2013) and recent investigations of ccf-mtDNA in MS, focusing on individuals alive at CSF biopsy, identified increased ccf-mtDNA copies in both relapsing-remitting MS (RRMS) (Varhaug et al., 2017)and PMS (Leurs et al., 2017); with elevated ccf-mtDNA appearing associated with brain atrophy (Leurs et al., 2017).

To date, there are no accurate predictors of neurodegeneration in MS. (Harris et al., 2017) The identification of markers which could inform neurodegenerative risk would be useful to both existing therapeutic strategies, but could also be used to inform future MS clinical trials (Harris et al., 2017). Given the links between MS progression and mitochondrial DNA (Campbell et al., 2011; Campbell and Mahad, 2018) and previous evidence linking ccf-mtDNA to other neurodegenerative diseases (Pyle et al., 2015a; Podlesniy et al., 2013), we analysed the ccf-mtDNA present in the post mortem ventricular CSF (vCSF) of PMS cases where neurodegeneration is more pronounced, comparing both ccf-mtDNA abundance and integrity to matched controls. In addition, we assessed the levels of known neurodegenerative disease (glial fibrillary acidic protein and S100 calcium binding protein B) (Petzold et al., 2003; Yardan et al., 2011; Avsar et al., 2012; Michetti et al., 2012) and PMS protein markers (Chitinase 3 like 1 and Chitinase 3 like 2) (Sellebjerg et al., 2017; Hinsinger et al., 2015), correlating protein levels to ccf-mtDNA copies in both PMS cases and controls. We hypothesised that, similar to other neurodegenerative diseases such as AD and PD, CSF ccf-mtDNA levels would be significantly lower in PMS cases with evidence of neurodegeneration, indicating that a reduction in ccf-mtDNA in the CSF is a component of neurodegenerative disease.

## 2 Materials and methods

### 2.1 Subjects

36 PMS (30% male, mean age at death 63 ± 12 years) and 43 matched control (54% male, mean age at death 78 ± 10 years) ventricular CSF (vCSF) samples were obtained from The UK MS Society Tissue Bank (Imperial College, London). All vCSF samples were prepared (i.e. to remove cells) as described previously (Pyle et al., 2015a; Mollenhauer et al., 2008). Briefly, vCSF was centrifuged (2000 *g* for 10 min) at room temperature, aliquoted into CSF collection tubes and stored upright at −80 °C. PMS was diagnosed in-life and confirmed neuropathologically (UK MS Society Tissue Bank, Imperial College, London). All controls were negative for the hallmarks of disease-related neurodegeneration or inflammation. Importantly, we saw no significant differences in age at death, brain weight post mortem interval or male:female ratio between PMS cases and controls, summarised in Supplementary Table 1.

### 2.2 mtDNA copy and deletion quantification

Mitochondrial DNA was quantified using established methods (previously described in (Pyle et al., 2015a; Grady et al., 2014; Pyle et al., 2016). Briefly, two mitochondrial regions, *MTND1*, *MTND4* (Supplementary Fig. 2) and a nuclear encoded housekeeping gene, *B2M*, were amplified and assayed in triplicate by multiplex Taqman mediated qPCR as per manufacturer's guidelines (ThermoFisher Scientific, primers: *MTND1* forward 5′-ACGCCATAAAACTCTTCACCAAAG-3′ and reverse 5′-GGGTTCATAGTAGAAGAGCGATGG-3′, *MTND4* forward 5′-ACCTTGGCTATCATCACCCGAT-3′ and reverse: 5′-AGTGCGATGAGTAGGGGAAGG-3′, and B2M forward 5′-CACTGAAAAAGATGAGTATGCC-3′ and reverse 5′- AACATTCCCTGACAATCCC-3′). As in previous work (Coxhead et al., 2016), mtDNA primer efficiency and specificity was assessed as accurate after zero amplification of DNA from Rho0 (mtDNA depleted) cell lines, avoiding the unintended amplification of nuclear pseudogenes. mtDNA level is calculated as an absolute measurement of *MTND1* derived from a triplicated standard curve and is expressed as copies per 1 μl of CSF. mtDNA deletion level, expressed as a ratio of *MTND1* to *MTND4* as described previously (Pyle et al., 2015a; Grady et al., 2014; Pyle et al., 2016). mtDNA deletions, as in previous work (Grady et al., 2014), were limited to those >10%; to account for the sensitivity and specificity of qPCR to reliably detect mtDNA deletions.

### 2.3 mtDNA deep-sequencing

Deep sequencing and subsequent bioinformatic analysis of mtDNA extracted from a subset of vCSF samples (12 PMS and 22 controls, 41% of samples) was performed as previously described (Coxhead et al., 2016). Briefly, DNA was extracted from vCSF using Ultrapure™ (phenol:chloroform:isoamyl alcohol) and ethanol precipitation as per manufacturer's instructions (SigmaAldritch). mtDNA was enriched using three overlapping long-range PCR amplicons covering the entire mtDNA genome (set 1, m.3016-9201, forward 5′-CAGCCGCTATTAAAGGTTCG-3′ and reverse: 5′-GTTGTCGTGCAGGTAGAGG-3′; set 2, m.8656-14,857, forward 5′-ACCACCCAACAATGACTAATC-3′ and reverse 5′-GGTTGTTTGATCCCGTTTCG-3′, and set 3, m.14797-3574, forward 5′-ATTCATCGACCTCCCCACC-3′ and reverse: 5′-GGAGGGGGGTTCATAGTAG-3′) and was amplified using PrimeSTAR GXL DNA Polymerase as per manufactures guidelines (Takara Bio Company). As in previous work (Coxhead et al., 2016), mtDNA primer efficiency and specificity was assessed as accurate after zero amplification of DNA from Rho0 (mtDNA depleted) cell lines, avoiding the unintended amplification of nuclear pseudogenes. In 59% (47 out of 81) of vCSF samples, mtDNA enrichment was hindered by low mtDNA template availability. Subsequent PCR products were purified, pooled and prepared using Illumina Nextera XT Library preparation kit using manufacturers guidelines (Illumina, USA). Libraries were pooled using the MiSeq Reagent Kit and the Illumina MiSeq v3.0 sequencing platform (Illumina, USA) in paired-end, 250 bp reads. Post-run FASTQ files were analysed using an established bioinformatic pipeline as previously described (Coxhead et al., 2016). Homoplasmic variation was defined as >99% Heteroplasmic variation was defined as >1% and < 99%. Variant comparisons, i.e. mutational burden were made between PMS case and control CSF ccf-mtDNA.

### 2.4 Protein quantification

Glial fibrillary acidic protein (GFAP) and S100 calcium binding protein B (S100β), previously used to indicate neurodegeneration (Petzold et al., 2003; Yardan et al., 2011), and PMS (Avsar et al., 2012; Michetti et al., 2012) were measured in all samples with sufficient vCSF (27 PMS and 15 controls, 51% of samples) using western blot, with protein levels normalised to total protein level measured with BLOT-FastStain™. Chitinase 3 like 1 (CHI3L1), used to differentiate active and inactive PMS (Sellebjerg et al., 2017), and Chitinase 3 like 2 (CHI3L2), used to differentiate PMS and RRMS (Hinsinger et al., 2015), were measured by ELISA (Cusabio, YKL-40 and R&D systems DC3 L10, respectively) in the same samples as above (i.e. 27 PMS and 15 controls) as per manufacturers guidelines.

### 2.5 Statistical analysis

Data was analysed using SPSS v22 and Prism v5 using data appropriate tests (detailed in the text). Statistical significance was set at *p* < .05. Heteroplasmic Ka/Ks is expressed as a ratio of non-synonymous (Ka) to synonymous (Ks) heteroplasmic variants (Li et al., 1985).

## 3 Results

a) Analysis of vCSF ccf-mtDNA in PMS

**Fig. 1:**
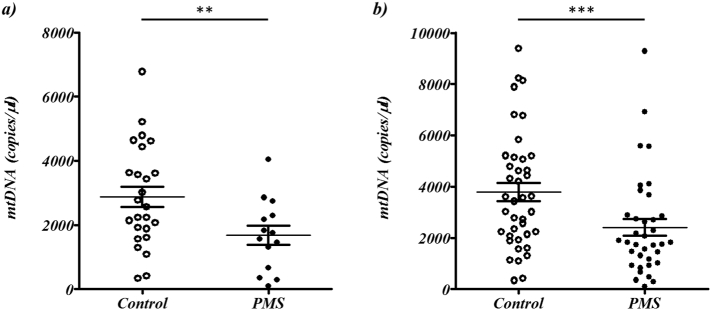
Comparison of ccf-mtDNA copies between PMS cases and controls. The plots depict the comparison of ccf-mtDNA copies (per μl vCSF) between PMS cases (shaded) and matched controls (unshaded): a) with a B2M threshold of <1 copy applied (where mean ccf-mtDNA copies in 14 PMS cases was 1678.0, SEM of 298.4, and 2876.2, SEM of 317.0, in 25 controls) and, b) without a B2M threshold applied (where mean ccf-mtDNA copies in 36 PMS cases was 2412.7, SEM of 327.5, and 3784.7, SEM of 356.4, in 43 controls), where ** and *** indicate Wilcoxon Ranked Sum Test significance at *p* < .05 and *p* < .005 respectively.

Similar to our previous work (Pyle et al., 2015a), and the work of others (Podlesniy et al., 2013; Varhaug et al., 2017; Leurs et al., 2017), we limited our analysis to samples harbouring <1 copy of a housekeeping gene (*B2M*) to minimise the perceived contamination of cells or cellular debris in the vCSF; reducing our cohort to 14 PMS cases (38%) and 25 controls (58%). Using these criteria we identified a significant reduction of vCSF ccf-mtDNA level in PMS cases compared to controls (Wilcoxon Ranked Sum Test *p* = .013, mean rank ccf-mtDNA in 14 PMS cases was 16.4 and in 25 controls was 26.9, Fig. 1a).

However, recent reports show evidence of cell free-nuclear DNA (ccf-nDNA) in the CSF (Adalsteinsson et al., 2017; Connolly et al., 2017), suggesting that nDNA contamination may not necessarily be a result of carryover of cells or a product of cellular debris during DNA extraction. In addition, the existing literature suggests that ccf-mtDNA resists degradation whilst ccf-nDNA does not (Manuelidis, 2011), raising the possibility that ccf-nucleic acids are still a consequence of cellular degradation, but whilst the ccf-nDNA has been degraded, the ccf-mtDNA has persisted. Therefore we removed the *B2M* threshold from our analysis, continuing to identify a significant decrease of vCSF ccf-mtDNA copies in PMS cases compared to controls (Wilcoxon Ranked Sum Test *p* = .0023, mean rank ccf-mtDNA in 36 PMS cases was 30.4 and in 43 controls was 45.8, Fig. 1b).

To investigate further, we tested the effect of *B2M* level on ccf-mtDNA level in PMS using logistic regression, confirming our previous association between PMS and ccf-mtDNA when including *B2M* level as a covariate (*p* = .004), identifying no significant correlation between *B2M* levels and ccf-mtDNA levels (r^2^ = 2.5 × 10^−7^ and *p* = 99, Supplementary Fig. 1) and no significant difference in *B2M* levels between PMS cases and controls (Wilcoxon Ranked Sum Test *p* > .05).

Given that *B2M* levels do not appear to effect ccf-mtDNA abundance, and to increase sample size and statistical power, all subsequent analysis will refer to the full dataset (36 PMS cases and 43 controls). Linear regression showed no significant association (*p* > .05) between ccf-mtDNA and age at death, brain weight, post mortem interval (in PMS cases, controls or combined) or age of onset and disease duration (PMS cases only), and we found no significant association (Logistic regression p > .05) between ccf-mtDNA and sex (in PMS cases, controls or combined, summarised in Supplementary Table 2).

b) ccf-mtDNA integrity in PMS vCSF

**Fig. 2:**
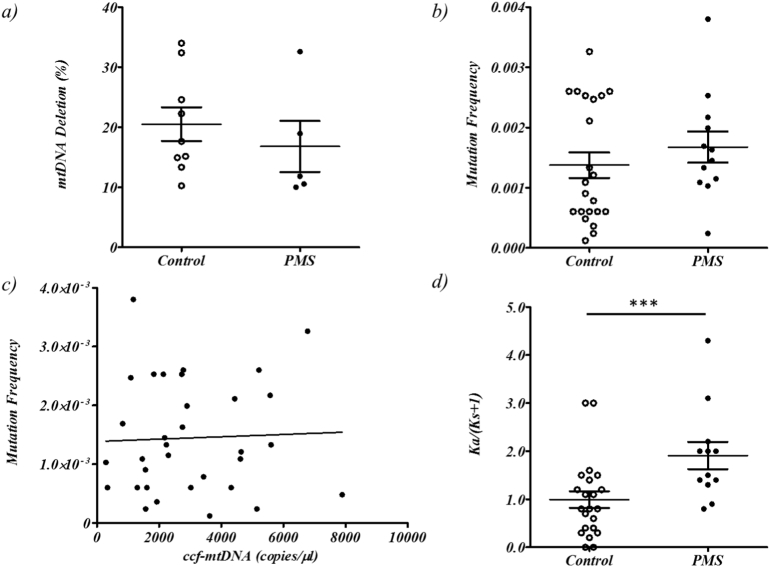
Analysis of ccf-mtDNA integrity in PMS cases and controls. The plots depict: a) comparative ccf-mtDNA deletions (>10%) detected in PMS cases and matched controls (mean deletion in 5 PMS cases was 16.8, SEM of 4.2, and 20.5, SEM of 2.8, in 9 controls), b) the comparative total heteroplasmic mutation frequency (heteroplasmy per bp sequenced) in PMS cases and controls (mean total heteroplasmic mutation frequency in 12 PMS cases was 2.6 × 10^−3^, SEM of 7.1 × 10^−4^, and 2.2 × 10^−3^, SEM of 4.3 × 10^−4^, in 22 controls). c) total heteroplasmic mutation frequency versus ccf-mtDNA copies for PMS cases and matched controls combined (12 PMS cases and 22 controls), and d) the comparative Ka/Ks for PMS cases and matched controls (mean Ka/Ks in 12 PMS cases was 1.9, SEM of 0.28, and 0.99, SEM of 0.17, in 22 controls), where *** indicates Wilcoxon Ranked Sum Test significance at *p* < .005.

mtDNA deletions have been observed in PMS neuronal tissue (Campbell et al., 2011). We were able to identify mtDNA deletions (>10% deleted mtDNA) (Grady et al., 2014) in a minority of our samples (5 out of 36, or 13%, PMS cases and 9 out of 43, or 20%, controls), indicating that ccf-mtDNA is intact in the majority of samples (Supplementary Fig. 2). However we found no significant difference in ccf-mtDNA deletion levels between PMS cases and controls (Wilcoxon Ranked Sum Test *p* > .05, Fig. 2a) and no significant correlation (p > .05) between ccf-mtDNA deletion level and age at death, brain weight, post mortem interval (in either PMS cases, controls or when combined) or age of onset (PMS cases only) (Supplementary Table 2).

Next generation sequencing of ccf-mtDNA identified heteroplasmic variation in all 34 vCSF samples used. Although mutation frequency appeared higher in PMS cases compared to controls, the comparison was not significant (Wilcoxon Ranked Sum Test *p* > .05, mean and standard deviation of total heteroplasmic mutation frequency in 12 PMS cases was 1.6 × 10^−3^ ± 9 × 10^−4^ and 1.4 × 10^−3^ ± 1 × 10^−3^ in 22 controls, Fig. 2b and Supplementary Table 3).

Further stratification by variant type (e.g. D-loop variants, tRNA variants etc) revealed an increase in non-synonymous variation (mean heteroplasmic non-synonymous mutation frequency in PMS cases was 1.1 × 10^−3^and was 7.4 × 10^−4^ in controls) and a decrease in synonymous variation (mean heteroplasmic non-synonymous mutation frequency in PMS cases was 5.x10^−4^and was 9.7 × 10^−4^ in controls) between PMS cases and controls, although no comparison reached statistical significance (*p* > .05, Supplementary Fig. 3 and Supplementary Table 3).

Under the hypothesis that ccf-mtDNA export may be a product of normal mtDNA maintenance, we compared total heteroplasmic variation frequency to ccf-mtDNA level, but found no significant correlation (p > .05, Fig. 2c). Further, we found no significant correlation between total heteroplasmic mutation burden and age at death, brain weight, post mortem interval (in either PMS cases, controls or when combined) or age of onset (PMS cases only), summarised in Supplementary Table 2.

We did identify a significant difference in heteroplasmy Ka/Ks ratio, a correction used as an estimate of the degree of evolutionary constraint (Li et al., 1985), between PMS cases and controls (Wilcoxon Ranked Sum Test *p* = .0022, Fig. 2d); with PMS ccf-mtDNA having a higher proportion of non-synonymous to synonymous variation compared to controls. However, we found no significant correlation (*p* > .05) between Ka/Ks ratio and ccf-mtDNA level in either PMS cases, controls or when combined and no correlation between Ka/Ks ratio and age at death, brain weight, post mortem interval (in either PMS cases, controls or when combined) or age of onset (PMS cases only), summarised in Supplementary Table 2.

c) ccf-mtDNA and neurodegeneration

**Fig. 3:**
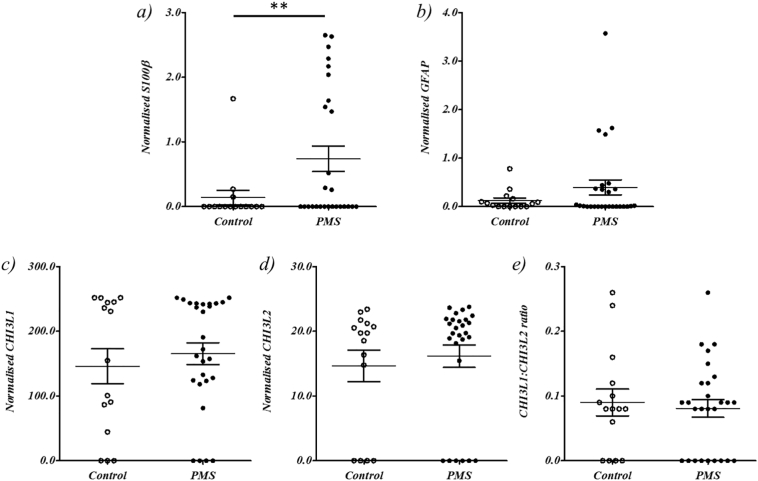
Comparison of neurodegenerative protein levels between PMS cases and controls. The plots depict the comparative levels of the neurodegenerative proteins S100β, GFAP, CHI3L1 and CHI3L2 (a to d) detected in 27 PMS cases and 15 controls and e) the relative ratio of CHI3L1 to CHI3L2 in PMS cases and controls, where ** indicates Wilcoxon Ranked Sum Test significance at *p* < .05.

Increased S100β is indicative of neurodegeneration (Rothermundt et al., 2003) and vCSF levels were significantly higher in PMS cases versus controls, (Wilcoxon Ranked Sum Test *p* = .040, mean and standard deviation in PMS cases was 0.99 ± 1.0 and 0.17 ± 0.48 in controls (Fig. 3a and Supplementary Table 4), with 14 out of 27 PMS cases (51%) and 12 out of 15 controls (80%) exhibiting undetectable levels.

Increased CSF levels of GFAP is indicative of neurodegeneration (Petzold et al., 2003) and increased levels have been observed on PMS patients (Avsar et al., 2012). We observed an increase of GFAP in PMS cases compared to control (Fig. 3b), although this did not reach statistical significance (Wilcoxon Ranked Sum Test *p* > .05, mean and standard deviation in PMS cases was 0.53 ± 0.0.90 and 0.16 ± 0.22 in controls), with 11 out of 27 PMS cases (41%) and 6 out of 15 controls (40%) exhibiting undetectable levels.

CSF CHI3L1 and CHI3L1 are elevated in PMS compared to controls (Hinsinger et al., 2015), with CHI3L1:CHI3L2 ratio reportedly having superior predictive accuracy than individual measures (Hinsinger et al., 2015). However, neither CHI3L1, CHI3L2 nor CHI3L1:CHI3L2 ratio were significantly different between PMS cases and matched controls when analysed together or as a ratio (Wilcoxon Ranked Sum Test p > .05, Figs. 3c-e and Supplementary Table 4).

However, ultimately, we saw no significant correlation between vCSF ccf-mtDNA and the levels of S100β, GFAP, CHI3L1, CHI3L2 or CHI3L1:CHI3L2 ratio when analysed as PMS cases only or controls only, or when combined (Supplementary Table 2).

## 4 Discussion

Our results indicate that vCSF ccf-mtDNA is significantly reduced in post-mortem PMS cases compared to controls which, when taken into context with observations in both Parkinson's disease (PD) (Pyle et al., 2015a) and Alzheimer's disease (AD) (Podlesniy et al., 2013), supports our hypothesis that ccf-mtDNA may be an component of neurodegeneration. After assessing mtDNA integrity, we conclude that ccf-mtDNA is largely intact, with few cases and controls harbouring mtDNA deletions. Interestingly we did observe a shift in the proportion of non-synonymous variation in the ccf-mtDNA of PMS cases compared to controls, which may be a product of ccf-mtDNA export, but which did not correlate to PMS or PMS-related phenotypic data. Ultimately, however, neither ccf-mtDNA level nor integrity correlated to protein markers of neurodegeneration.

Despite our observations, and an increasing amount of literature reporting associations between ccf-mtDNA levels and disease (>25 publications since 2014, PubMed keyword search using combinations of ‘cell’ ‘free’ ‘mitochondrial’ ‘DNA’ and appropriate abbreviations), we know little of the underlying causes of mtDNA export to intracellular spaces, therefore we opted to study the integrity of the ccf-mtDNA, contrasting PMS cases to controls.

It is possible that, assuming that mtDNA export is a normal procedure (as it occurs in control samples and typically at higher levels) (Pyle et al., 2015a; Podlesniy et al., 2013; Melentijevic et al., 2017), the lower levels seen in neurodegenerative disease is a direct consequence of a pre-existing cellular mtDNA depletion in vulnerable brain regions. This hypothesis is supported by previous studies showing tissue specific depletion of mtDNA in PD patients, (Pyle et al., 2015b; Grunewald et al., 2016) AD patients (Rice et al., 2014; Rodriguez-Santiago et al., 2001), and MS patients (Mao and Reddy, 2010b; Dutta et al., 2006; Ban et al., 2008; Blokhin et al., 2008a); indicating that low ccf-mtDNA observed in the vCSF of these cases is possibly a result of neuronal mtDNA levels which are already compromised prior to export.

Alternatively, it is possible that the release of mtDNA into intracellular spaces is a quality control mechanism, where mutant mtDNA is expelled to maintain mitochondrial function. This hypothesis is supported by recent neuronal work in *C. elegans*, which expel dysfunctional mitochondria when exposed to neurotoxic stress (Melentijevic et al., 2017). Further, the ependymal epithelium of the ventricles and choroid plexus have abundant mitochondrial content due to the high energy demand of transepithelial transport (Cornford et al., 1997). The majority of these mitochondria are located at the apical brush border (Spector et al., 2015), the most likely site of expulsion of whole mitochondria or cell-free mtDNA into the CSF during normal cell turnover or through active vesicular transport. Our data appear to support this hypothesis in part. Our investigations indicate that vCSF ccf-mtDNA is predominantly intact, with few samples showing measurable deletion levels, and although NGS analysis indicates the presence of heteroplasmic variation, we observed a non-significant increase in total heteroplasmic mutation and non-synonymous burden between PMS cases and controls. We did observe an imbalance in the Ka/Ks ratio between PMS cases and controls, with PMS cases exhibiting a greater than expected ratio of non-synonomous:synonymous variation, which may indicate that ccf-mtDNA export may be a selective process; where the ‘wild-type’ mtDNA molecule is retained in the cell and the ‘mutant’ mtDNA molecule is preferentially exported. This hypothesis supported by recent work in MS indicating that the neuronal tissues of cases harbour relatively few mtDNA mutations compared to matched controls (Blokhin et al., 2008b),although further work would be needed to be definitive.

Contrary to our findings, increased levels of ccf-mtDNA have been observed in the CSF of both RRMS (Varhaug et al., 2017) and PMS (Leurs et al., 2017). This is perhaps not unexpected in RRMS, which is an acutely inflammatory disease which precedes the neurodegenerative processes characteristic of PMS (Kingwell, 2009). It is possible therefore, that the increase in ccf-mtDNA observed in RRMS is a direct result of an increase in inflammatory cells which, in addition to nuclear DNA, release mtDNA into the CSF (Frank, 2016).

Leur's et al. (2018) (Leurs et al., 2017) report an increase of lumbar CSF (lCSF) ccf-mtDNA in PMS cases compared to controls. Given the parity in cohort sizes (40 PMS and 23 controls to our 36 PMS and 43 controls) the discrepancy between our results is unlikely to be a factor of statistical power. It is possible that the difference in CSF sampling may explain the discordance between results, with their samples taken in-life and ours taken post-mortem, and as the composition of vCSF may be different from lCSF in normal circumstances and under severe pathological conditions (Gerber et al., 1998; Sommer et al., 2002). However we observed no correlation between post mortem interval or brain weight to ccf-mtDNA levels in either PMS cases, controls or the cohorts combined, suggesting that the latter is not the case. Further, the increase in PMS ccf-mtDNA reported by Leurs et al. (2018) (Leurs et al., 2017) is limited to a small proportion of PMS cases (~30%) and the majority of PMS cases and controls exhibited ccf-mtDNA copies below the levels we observed in our study and other previously published lCSF mtDNA levels (typically 20–400 copies per microliter CSF in cases and 20–300 copies per microliter in controls in previous reports) (Podlesniy et al., 2013; Melentijevic et al., 2017; Pyle et al., 2015c). Our overall comparably higher ccf-mtDNA levels could be attributed to the increased autolysis or pleoctytosis which occurs in post-mortem CSF (Morris et al., 2012; Bardale, 2009), however we observed a significant reduction in ccf-mtDNA copies in PMS cases, despite no significant difference in PMI, brain weight or B2M levels between PMS cases and controls and no correlation between ccf-mtDNA and ccf-B2M levels, suggesting that this is not the case. It should be noted that, unlike Leurs et al. (2018) (Leurs et al., 2017), we were unable to assess the effect of ccf-mtDNA on brain atrophy directly.

With the exception of S100β, protein markers of neurodegeneration and PMS appeared uninformative when comparing cases and controls. Although GFAP and CHI3L1 appeared elevated in PMS cases compared to controls, the differences did not reach statistical significance. Further, we observed no obvious stratification of PMS cases using CHI3L1:CHI3L2 ratio, further indication that our cases were indeed all PMS cases (Hinsinger et al., 2015).Never the less, we observed no correlation between protein markers of neurodegeneration and ccf-mtDNA level or integrity; although this may reflect the late disease stage of our cases and the use of post-mortem CSF.

## 5 Conclusion

In conclusion, and excepting limitations, our findings support our hypothesis that decreased ccf-mtDNA may be an indicator of neurodegeneration in PMS. Although we observed a significant association, we urge caution. In light of previous work (Varhaug et al., 2017; Leurs et al., 2017), we cannot exclude the possibility that ccf-mtDNA levels rise in-life in response to the onset of disease and then reduce as neurodegeneration increases, however longitudinal assessments in PMS cases would be needed to assess this. Therefore, whilst reduced ccf-mtDNA may be a hallmark of the presence of neurodegeration, it is not particularly useful as a biomarker, even with a more detailed analysis.

## Author contributions

HL and AP performed the quantitative PCR and protein assessments in vCSF and were all involved in the design of the experiments and subsequent analysis. GH provided the scientific questions, contributed to the experimental concept and design and supervised all experimental aspects of this study, analysis of results, and with the assistance of MD, wrote and edited the manuscript.

## Potential conflicts of interest

The authors declare that they have no competing financial interests.

Correspondence and requests for materials should be addressed to GH: (Gavin.Hudson@ncl.ac.uk).
