# Supplementary material for: Cell-free mitochondrial DNA in progressive multiple sclerosis
Source: Mitochondrion. 2019 May;46:307–12. doi: 10.1016/j.mito.2018.07.008 (PMC6509276; doi:10.1016/j.mito.2018.07.008)

**Supplementary Figures**

**Supplementary Figure 1.**

Correlation of ccf-mtDNA levels to nDNA levels (B2M) in PMS cases and controls combined (36 and 43 respectively, totalling 79 samples). Linear regression shows no correlation, R^2^= 2.5x10^-7^ and p>0.05.


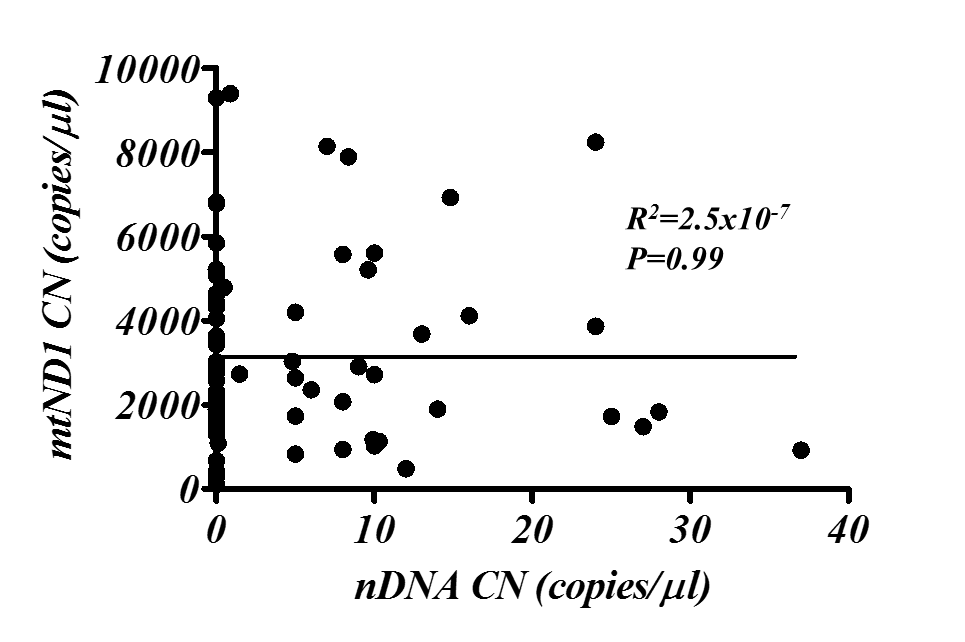


**Supplementary Figure 2.**

**a)** Cartoon of relative mtDNA amplicon positions (unshaded boxes) in the mtDNA molecule and **b)** correlation of MTND1 and MTND4 derived ccf-mtDNA copy number in PMS cases and controls combined (36 and 43 respectively, totalling 79 samples). Linear regression shows strong correlation, R^2^=0.798 and p=6.3x10^-18^.


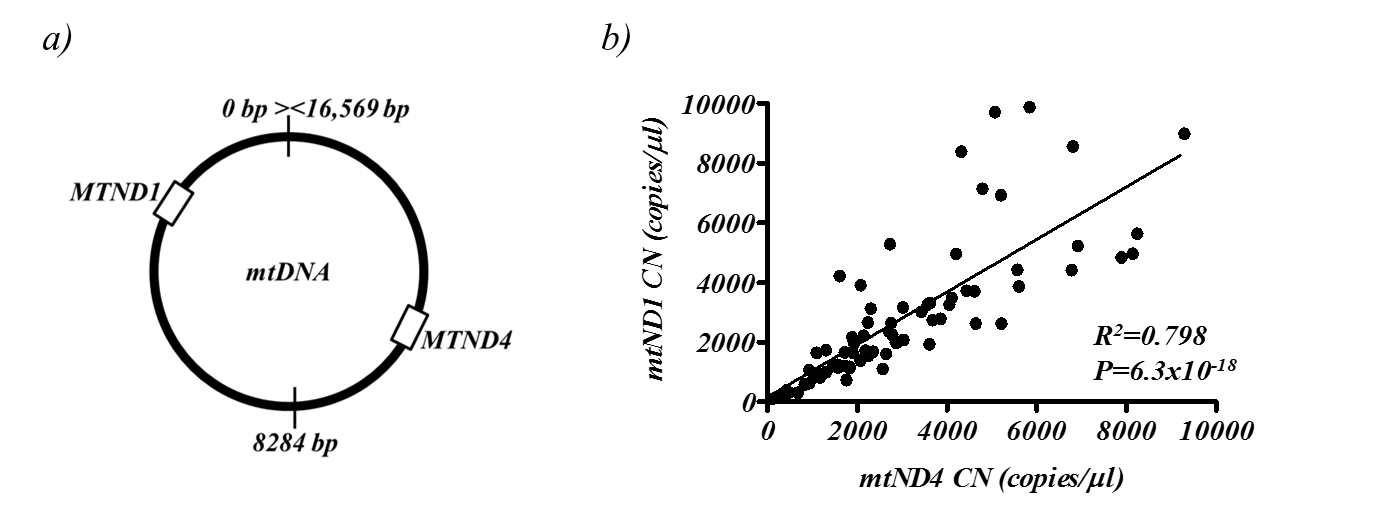


**Supplementary Figure 3.**

Plots of mean mutational burden in PMS cases (shaded) and controls (unshaded) (12 and 22 samples respectively) sub-stratified by mtDNA locus, e.g. D-loop variants only, rRNA variants only, tRNA variants only and total protein coding variants only (a to d). Protein coding mutational burden is further stratified into non-synonymous (Non Syn) and synonymous (Syn) variants only (e and f).


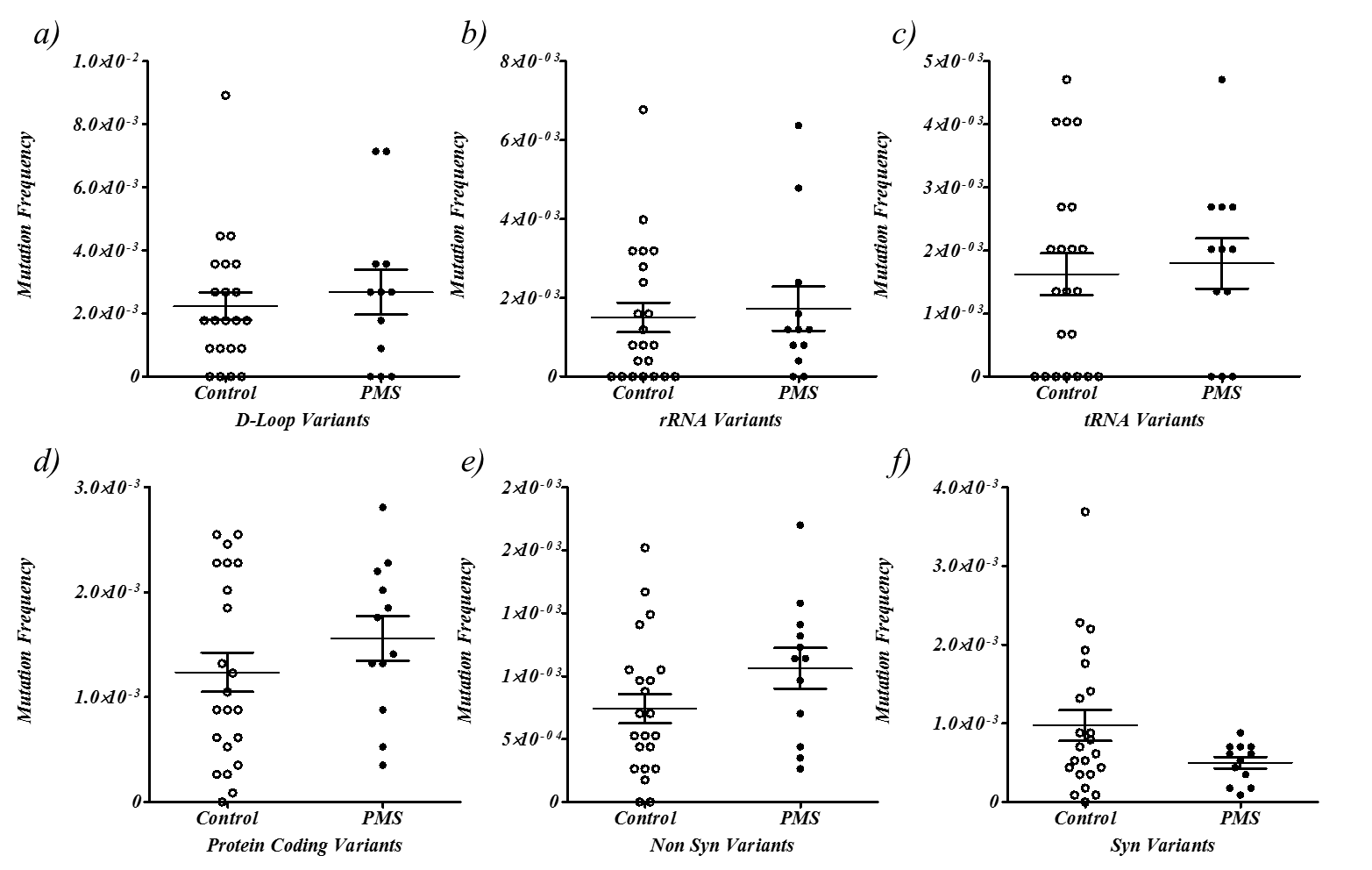

Supplement: Supplementary figures [file mmc1.docx]
